# A Lightweight Model for Hot-Rolled Steel Strip Surface Defect Recognition

**DOI:** 10.3390/s26051618

**Published:** 2026-03-04

**Authors:** Naixuan Guo, Haonan Fan, Qin Dong, Rongchen Gu, Sen Xu

**Affiliations:** 1 School of Information Engineering, Yancheng Institute of Technology, Yancheng 224051, China; 2 Jiangsu Provincial Engineering Technology Center for Multimodal Perception and Intelligent Control of Offshore Wind Power Systems, Yancheng 224051, China; 3 College of Underwater Acoustic Engineering, Harbin Engineering University, Harbin 150001, China

**Keywords:** defect recognition, image classification, convolutional neural network, data augmentation, network slimming

## Abstract

With the rapid development of intelligent manufacturing and industrial automation, defect recognition and detection of hot-rolled strip steel have become crucial to ensuring both production efficiency and product quality. However, existing hot-rolled strip steel detection systems often rely on expensive, energy-intensive, stationary equipment, making them unsuitable for mobile applications, such as outdoor use. To address this challenge, this paper proposes and designs a lightweight dual-surface defect recognition model for hot-rolled steel strips that can be implemented on mobile low-power devices (e.g., Raspberry Pi). First, to train the lightweight model, the NEU-CLS dataset is augmented through image generation via StyleGAN3, denoising with a water-wave-like noise removal algorithm, and super-resolution with Real-ESRGAN. Then, MMAM-EfficientNet-B0 is pruned during training, and the Network Slimming algorithm is applied to optimize it on the expanded NEU-CLS dataset, removing 70% of the network structure. Finally, the pruned recognition model is deployed on a Raspberry Pi, achieving an accuracy of 96.333%, with a classification time of 1.527 s per image, a reduction of 155.010% compared to the original model. Our experiments confirm the real-time effectiveness and practical application value of the model.

## 1. Introduction

With the development of the global economy, steel enterprises are shifting from increasing production capacity to intelligent and refined production, in which defect detection and recognition on the surface of hot-rolled steel strips (HRSSs) is a key technology to improve the quality of steel products and realize intelligent production [1]. At present, the production line speed is gradually increasing, the user’s product quality requirements are increasingly strict, and there is an urgent need to improve the detection efficiency and recognition accuracy of surface defects in strip steel. However, existing defect detection and recognition methods often rely on expensive GPUs with high computational power. These devices are usually stationary, consume a lot of energy, and are not suitable for detection in mobile or no-power-supply scenarios. Therefore, it is necessary to study a more lightweight model that can run in embedded devices, meeting the mobility requirements of industrial production while ensuring accuracy.

Among the many HRSS classification models proposed, most of them focus on improving classification accuracy [2,3,4]. Among these approaches, few scholars have ported HRSS classification algorithms to the embedded end, making them ineffective for mills. The obsession with accuracy will inevitably make the network deeper and the model bloated, which further leads to excessive energy consumption during operation. To solve this problem, many scholars have proposed different methods. Li et al. [5] proposed a lightweight network, CASI-Net, which can effectively reduce the parameters through the design of lightweight modules in the network; unfortunately, the authors did not port the algorithm to the embedded side for further research. Lu et al. [6] used knowledge distillation to improve the performance of the model by using a larger training network to teach a smaller network, and obtained high accuracy using only 0.03 MB of parameters. Although the article states that it can be applied to small mobile devices, it is not put into practice. Currently, research in this area faces the following shortcomings:

1. There is a lack of a light and convenient HRSS dual-surface defect detection and recognition model with high efficiency and accuracy;
2. There are insufficient samples in the commonly used NEU-CLS dataset for HRSS surface defects, resulting in subpar performance when used for training pruned models [7];
3. Model pruning algorithms have rarely been applied to HRSS surface defect classification methods.

To compensate for the above shortcomings, this paper proposes a lightweight model for detecting and recognizing dual-surface defects in HRSS. The MMAM-EfficientNet-B0 model [8] is chosen for the basic model of the recognition algorithm, and the model is pruned and improved. Before pruning training, we perform data augmentation on the NEU-CLS dataset to expand 1800 images to 6000 images using StyleGAN3 [9] to generate the images, the water-wave-like noise removal (WWNR) algorithm to denoise them, and Real-ESGAN [10] to repair them with super-resolution. In the pruning process, we remove the squeeze-and-excitation (SE) module from MMAM-EfficientNet-B0, prune the original model to 70% of its original size using the Network Slimming [11] method, and obtain an accuracy of 99.875% in the expanded dataset. The pruned recognition algorithm ported to Raspberry Pi obtains a 96.333% accuracy in the original NEU-CLS dataset, and the recognition classification time for a single image is much less compared to the original model.

In summary, the main contributions of this work are:

1. A lightweight HRSS dual-surface defect detection model with high efficiency and accuracy is proposed and designed;
2. Combination of StyleGAN3-generated images, WWNR algorithm denoising, and Real-ESGAN super-resolution restoration of images, and used for data augmentation of the NEU-CLS dataset;
3. A pruned and modified for the MMAM-EfficientNet-B0 model is proposed, and we apply it to the Raspberry Pi.

The remainder of this paper is organized as follows. Section 2 introduces related work. Section 3 presents the design of our algorithm model. Section 4 describes the experimental process and analyzes the experimental results. Section 5 discusses and summarizes the methods employed in this paper.

## 2. Related Works

Given that this paper focuses on HRSS data augmentation and lightweight classification models, this section provides an overview of representative and relevant studies in these two research directions.

### 2.1. Data Augmentation Based on Generative Adversarial Networks

In 2014, Ian Goodfellow et al. [12] combined a restricted Boltzmann machine with a variational autoencoder, introduced the idea of maximal and minimal bilateral games, and proposed a generative adversarial network model consisting of a discriminator and generator to form a generative adversarial network (GAN). GAN was proposed for generating realistic images, and later, many scholars improved and optimized this architecture and used it for the augmentation of different types of data.

Cui et al. [13] applied GAN to Synthetic aperture radar (SAR) image data augmentation by utilizing Wasserstein GAN (WGAN-GP) to increase the number of samples in the training dataset based on the existing SAR data. Based on this, filters are added to extract high-quality and specific azimuthal angles from the generated samples to avoid randomness in data augmentation and to improve the quality of the newly generated training samples. Gao et al. [14] proposed a leaf-bootstrapping method to improve the performance of DCGAN (Deep Convolutional Generative Adversarial Network) under the opportunity of a lack of labeled data in Civil Engineering problems. Mok et al. [15] applied data augmentation with GAN to the medical imaging field, using the data augmentation method of GAN to address the challenge of deep neural network training difficulties due to limited training samples for brain tumor segmentation containing annotations. The application of GANs for data augmentation effectively addresses the problem of insufficient training samples in medical imaging [16]. Sandfort et al. [17] applied CycleGAN to the task of visceral CT segmentation, which reduces the manual segmentation effort and cost in CT imaging. Liu et al. [18] proposed a Leaf GAN model, which provides a feasible solution for data augmentation of grape leaf disease images, improves the recognition accuracy, and overcomes the overfitting problem faced by the recognition models.

The generative ability of GANs is not limited to images; many scholars have also used the method to generate other types of data, such as speech, signals, and so on. Qian et al. [19] used a GAN to generate speech samples by generating spectral features based on spectral features applied to a noisy task and got significant improvement. Shao et al. [20] obtained convincing sensor data by applying the framework of ACGAN for data augmentation in machine fault diagnosis of mechanical sensor signals machines. Gao et al. [21] applied WGAN-GP to the challenge of low data or unbalanced datasets in industrial fault diagnosis to generate data samples that complement the low data input set in the field of fault diagnosis and contribute to the improvement of fault diagnosis accuracy. Fahimi et al. [22] introduced a DCGAN-based framework for generating artificial electroencephalography (EEG) data to enhance the performance of brain-computer interface (BCI) classifiers. The proposed method achieved promising results in generating subject-specific artificial EEG data to reduce the calibration time in EEG-based BCI applications.

### 2.2. Research on Lightweight Surface Defect Classification Algorithms for HRSS

With advances in graphical computing and deep learning, the classification of surface defects in strip steel has gradually shifted from manual methods to computer vision. Lightweighting of the model is essential if the proposed visual model is to be applied in practice.

Zhou et al. [23] lightened YOLOv5s by using the Ghost module, which was used to replace parts of the network’s structure to reduce the model parameters and complexity, thus reducing the model’s parameters and computation and improving efficiency. The experimental results prove that the proposed model meets the demand for real-time recognition of HRSS defects in industrial production. Bouguettaya et al. [24] selected two state-of-the-art MobileNet-V2 and Xception lightweight models as the infrastructure and combined them with transfer learning for training. The selection of these lightweight models improves the speed of model recognition and helps in the real-time development of a surface defect recognition system for HRSS. The following year, they [3] compared the then state-of-the-art convolutional neural network (CNN) models in terms of classification performance by screening them on the NEU-CLS dataset, and concluded that the methods based on MobileNet-V2 and InceptionResNetV2 outperformed the other models in terms of accuracy, loss, training and inference time, and model size. Shao et al. [25] proposed a multi-scale lightweight neural network model for the classification of surface defects of steel bars, which takes the fusion coding module as the core and utilizes the Gaussian difference pyramid to construct a multi-scale neural network, which not only reduces the number of model parameters but also compresses the model size and achieves better classification accuracy.

In addition to lightweighting the model itself, there are other ideas to improve the efficiency of strip inspection. Luo et al. [26] proposed selectively dominant local binary patterns (SDLBPs) for the classification of defects, which requires less computing power. For surface defect detection, the SDLBP framework achieves a balanced performance between classification accuracy and time efficiency, with the flexibility to obtain various variants depending on the application. Zhang et al. [27] are to remove spatial redundancy, coding redundancy, and extraneous information redundancy from an image by image compression, thus reducing the storage space of the image and improving the transmission efficiency. The method uses a Convolutional Autoencoder for image compression preprocessing to remove noise unrelated to defects in the detection of defects on the surface of HRSS, which improves the accuracy and efficiency of defect detection.

In summary, although some researchers focus on the lightweight model itself and claim the method can be used on mobile devices, few scholars have conducted tests on mobile devices.

## 3. Recognition Algorithm Model Optimization

In this section, we will introduce the model optimization of the HRSS surface defect recognition algorithm. We describe the overall structure of the model optimization,  then introduce data augmentation and model pruning. In data augmentation, this is achieved through StyleGAN3 image generation, WWNR denoising, and Real-ESGAN super-resolution. In model pruning, the MMAM-EfficientNet-B0 model is pruned using the newly generated dataset and the Network Slimming algorithm [11] to achieve model compression.

### 3.1. Overall Structure of the Model Optimization Method for Recognition Algorithm

**Figure 1:**
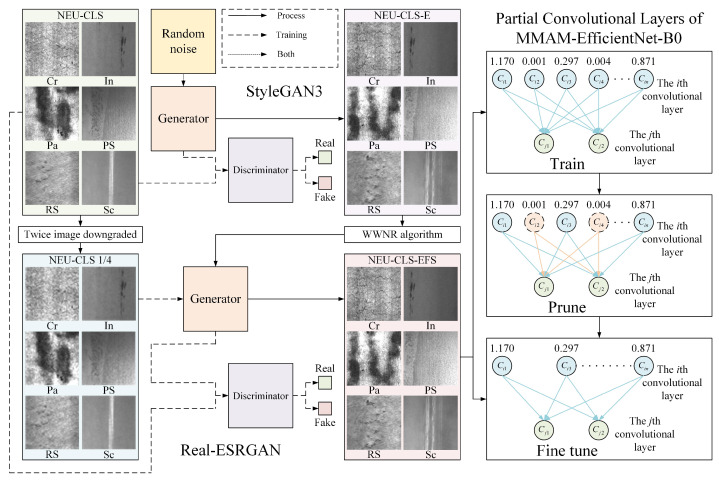
Overall flowchart of the model optimization method for recognition algorithm.

The recognition algorithm we used is based on MMAM-EfficientNet-B0, data expansion by GAN, and network compression by Network Slimming. The overall flowchart is shown in Figure 1.

To realize MMAM-EfficientNet-B0 for better and faster recognition of surface defects on HRSS at the embedded end, network pruning is performed by the Network Slimming method, and the data required for network pruning is expanded by a combination of StyleGAN3 [9] and Real-ESRGAN [10]. We import the original dataset NEU-CLS into the StyleGAN3 model for training. After the training is completed, the data named NEU-CLS-E is obtained by random vectors brought into the generator of the GAN. The NEU-CLS dataset will be described in detail in Section 4.1.1. There is a lot of water-wave-like noise in NEU-CLS-E. To remove the effect of these noises on the model compression training, the WWNR algorithm is utilized for denoising, and then super-resolution computation is carried out through the Real-ESRGAN model, and the size is adjusted to obtain the final expanded dataset NEU-CLS-EFS. The NEU-CLS-EFS contains much more data than the original dataset, enabling better results in the Network Slimming model training [7]. In model pruning, the compression of the network is achieved through pre-training, pruning, and fine-tuning to obtain the final network structure and weights, where both pre-training and fine-tuning are performed on the NEU-CLS-EFS dataset.

### 3.2. Data Augmentation

We first perform data augmentation with StyleGAN3 and discover the problem of waterline noise that can arise. Fan et al. [8] Clarifies the limitations of traditional data augmentation methods, and emphasizes the core advantages of the proposed augmentation method combining Generative Adversarial Networks (GAN) in terms of defect feature expansion and sample authenticity. To address this problem, we propose the WWNR algorithm to remove the waterline noise, and then use the Real-ESRGAN super-resolution method to repair the removed image details, which ultimately realizes the data enhancement.

#### 3.2.1. StyleGAN3 Data Augmentation

**Figure 2:**
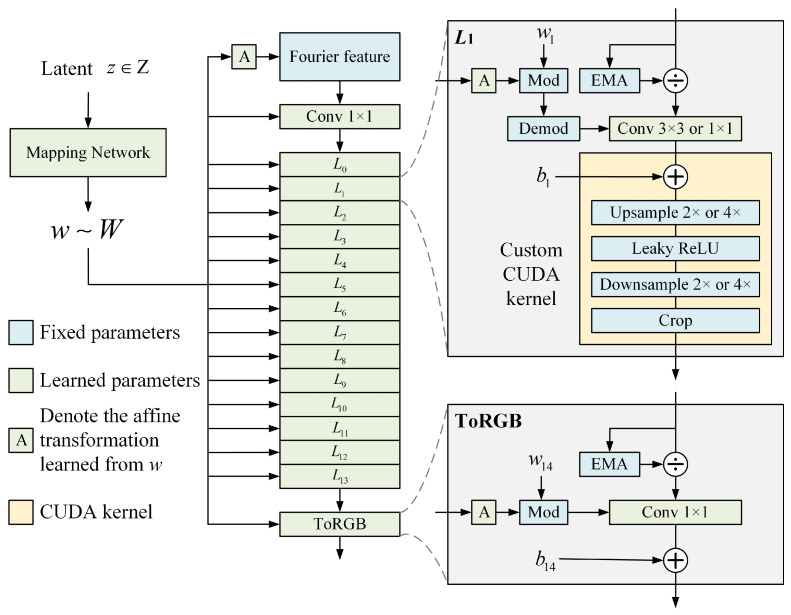
The structure of StyleGAN3 generator [9].

We apply StyleGAN3, a face-expansion model, to the HRSS surface defects dataset, and the images trained on the NEU-CLS dataset are more detailed and lifelike than those from other datasets. The unique feature of this model is the generator network, as shown in Figure 2.

We follow the method’s guidelines and divide the generator into two sub-networks: the mapping network and the synthesis network, whose roles are to generate diversification parameters and images, respectively. In the mapping network, the input is a latent z that conforms to a uniform or Gaussian distribution, so the coupling between the variables is relatively large, and the mapping network is needed to decouple z into w, which is then fed into the synthesized network through affine transformations, respectively. In the synthesis network, a 13-layer style block adapts the input features according to w, thus affecting the direction of final image generation.

**Figure 3:**
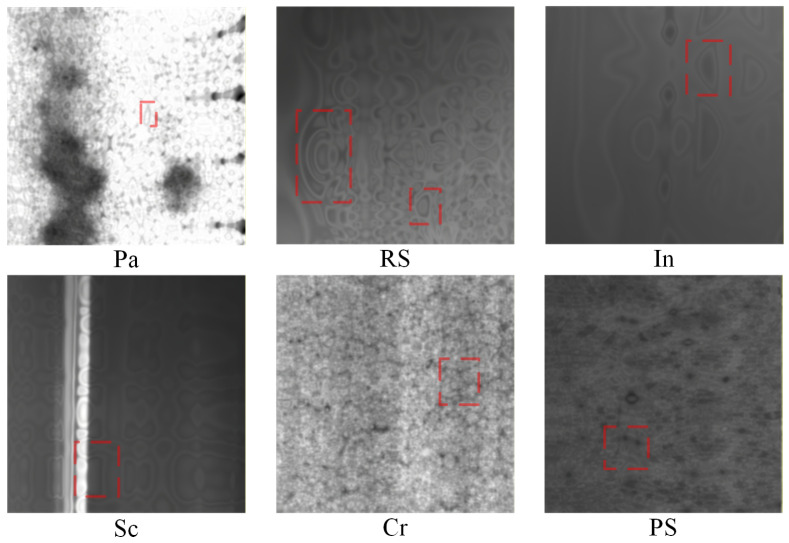
Water-wave-like noise in generated images.

The key improvement of StyleGAN3 over its predecessor is the mapping of discrete features to continuous features, which allows Fourier features to be easily subjected to translation and rotation operations, resulting in more realistic and detailed images. But this also consequently appears as a water-wave-like noise in the generated image, as shown in Figure 3. The more representative water-wave-like noise is marked out in red.

#### 3.2.2. Water-Wave-like Noise-Removal Algorithm

To minimize the effect of water-wave-like noise in the generated image, we propose a water-wave-like noise-removal (WWNR) algorithm. Based on the properties of water ripples, we found the center point of the ripples through morphological operations. Morphological operations can be used to thin and connect the water wave by an erosion operation and then to find the center of the water wave by an expansion operation. Once a candidate region for the centroid of the water wave has been identified, the least-squares method can be used to further optimize the centroid’s location and to use it as the center to form a mask in preparation for later filtering and noise reduction.

Bilateral filtering is chosen as a filtering and noise-reduction method that can preserve edges while smoothing noise, making it more suitable for removing water-wave-like noise. Like other filtering principles, such as mean filtering, bilateral filtering uses weighted averaging to represent the intensity of a particular pixel as a weighted average of the surrounding pixel brightness values, and the weighted average used is based on a Gaussian distribution. In bilateral filtering, the weights take into account not only the Euclidean distance of the pixels, but also the radial differences in the pixel range domain, such as the degree of similarity between the pixels in the convolution kernel and the center pixel, the color intensity, and the depth distance. These two weights are considered simultaneously in the calculation process as shown in Equation (1):

(1) $$I_{p}^{\mathrm{bf}}=\frac{1}{W_{p}^{\mathrm{bf}}}\sum_{q\in S}G_{\sigma_{s}}\left(∥p-q∥\right)G_{\sigma_{r}}\left(\left|I_{p}-I_{q}\right|\right)I_{q},$$

where the normalization factor $W_{p}^{\mathrm{bf}}$ is as follows:

(2) $$W_{p}^{\mathrm{bf}}=\sum_{q\in S}G_{\sigma_{s}}\left(∥p-q∥\right)G_{\sigma_{r}}\left(\left|I_{p}-I_{q}\right|\right).$$

In the above two equations, $I_{p}^{\mathrm{bf}}$ denotes the value of pixel *p* after bilateral filtering, $I_{q}$ is the value of pixel *q*, and *S* denotes the window centered at *p*. $G_{\sigma_{s}}$ is a spatial domain kernel that depends on the distance of the pixel position. $G_{\sigma_{r}}$ is a pixel domain kernel that depends on the difference in pixel values. In the flat region of the image, the pixel values vary less, and the corresponding pixel range domain weights are close to 1. At this time, the spatial domain weights play a dominant role, and the Gaussian blurring effect is realized. Whereas, in the edge region of the image, the pixel values vary more, and the pixel range domain weights are increased so that the edge information is preserved.

In Equations (1) and (2), the spatial domain kernel is calculated as follows:

(3) $$G_{\sigma_{s}}\left(∥p-q∥\right)=e^{-\frac{{\left(i-m\right)}^{2}+{\left(j-n\right)}^{2}}{2{\sigma_{s}}^{2}}},$$

The equation for the pixel domain kernel is as follows:

(4) $$G_{\sigma_{r}}\left(\left|I_{P}-I_{q}\right|\right)=e^{-\frac{{\left[I_{\left(i,j\right)}-I_{\left(m,n\right)}\right]}^{2}}{2{\sigma_{r}}^{2}}},$$

In Equations (3) and (4), $\sigma_{s}$ and $\sigma_{r}$ are set parameters, i,j,m and *n* are coordinate position variables during the computation process, (i,j) represents the center of the window p,(m,n) represents a value *q* in the sliding window.

**Figure 4:**
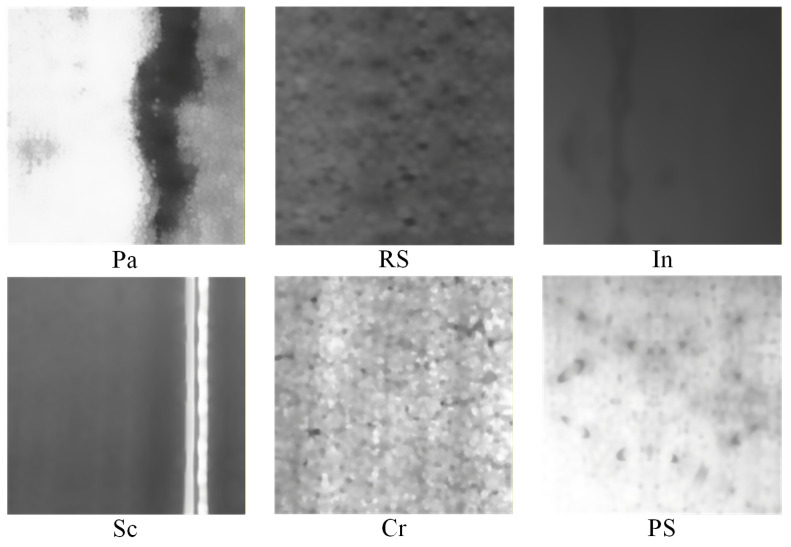
WWNR algorithm denoising effect.

The water-wave-like noise removal method for HRSS defective images generated by SyleGAN3 is shown in Algorithm 1. The WWNR algorithm removes most of the water-wave-like noise, but it also mistakenly removes some details of the strip defects, which can have an impact on the subsequent model training, as shown in Figure 4.

**Algorithm 1:** Water-Wave-like Noise-Removal Algorithm

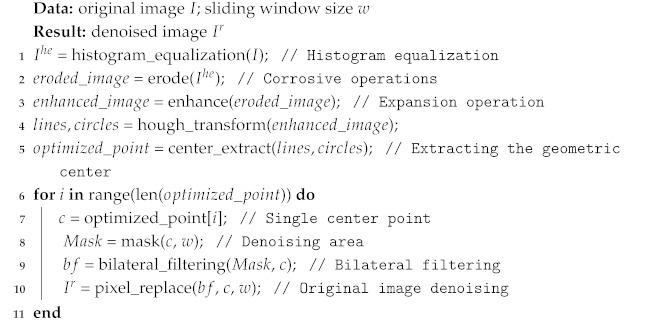



#### 3.2.3. Real-ESRGAN Super-Resolution

To compensate for the loss of details brought about by the WWNR algorithm on the generated images, we employ the Real-ESRGAN network to repair and refine the details. We self-construct the NEU-CLS high-resolution, low-resolution paired dataset. The Real-ESRGAN network is then trained on this data to obtain the generator’s training weights. Then the filtered and denoised image is imported into the Real-ESRGAN generator, super-resolved to a high-resolution image. The final image is compressed to its original resolution.

**Figure 5:**
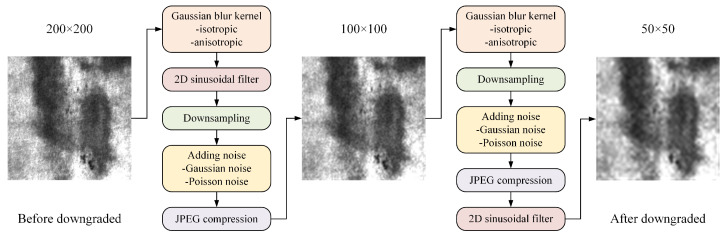
Twice one-level image downgraded.

The self-constructed NEU-CLS super-resolution dataset is obtained by taking the original NEU-CLS dataset as the high-resolution data and performing two first-order degradations [10] on this data to obtain pairs of low-resolution data, as shown in Figure 5. The first-order degradation model is the conventional degradation model, as shown in Equation (5):

(5) $$x=D\left(y\right)={\left[\left(y⊗k\right){↓}_{r}+n\right]}_{\mathrm{JPEG}},\;$$

where *x* denotes the degraded image, *D* denotes the degradation function, *y* denotes the original image, *k* denotes the blur kernel, *r* denotes the reduction ratio, *n* denotes the added noise and JPEG denotes the compression performed. For the Gaussian blur kernel, isotropic and anisotropic kernels are included. The purpose of adding a 2D sinusoidal filter is mainly to set different parameters to mimic the ringing and overshoot artifacts in practice. For downsampling, we use bilinear interpolation to realize the image reduction operation. The added noise obeys not only the Gaussian distribution but also the Poisson distribution. JPEG compression reduces the size of image files while maintaining high image quality.

**Figure 6:**
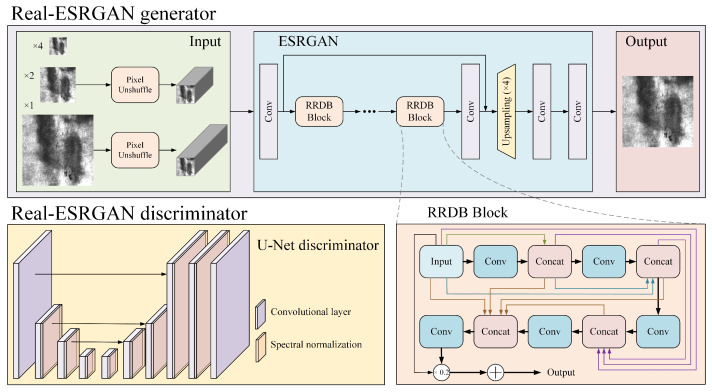
The structure of Real-ESRGAN.

The Real-ESRGAN network has the same basic structure as a GAN and consists of two parts, the generator and the discriminator, as shown in Figure 6. The generative network is ESRGAN, which adds image sharpness enhancement with scaling factors of ×2 and ×1 to the original. For the super-resolution of the input image with a scaling factor ×4, the network is fully consistent with the ESRGAN generator structure; For the super-resolution of the input image with scaling factors ×1 and ×2, the incoming feature maps are first pixel-unshuffled. Here, pixel-unshuffle is the inverse operation of pixel-shuffle, i.e., the tensor with shape $\left(n,\;C,\;H\times r,\;W\times r\right)$ is rearranged and converted to a tensor with shape $\left(n,\;C\times r^{2},\;H,\;W\right)$. The number of image channels is expanded to reduce image resolution, and the processed image is then fed into an ESRGAN network comprising a convolutional layer, a Residual-in-Residual Dense Block (RRDB) module, an upsampling layer, etc., for super-resolution reconstruction. The discriminative network is selected from U-Net’s discriminative network [28], which is capable of judging the authenticity of individual generated pixels, ensuring that the details of the generated image are attended to while the generated image as a whole remains authentic. The network mainly consists of convolutional layers and spectral normalization. The introduction of spectral normalization helps to alleviate the training instability problem caused by complex datasets and network structures.

### 3.3. Model Pruning

We use channel-wise pruning methods to achieve model sparsity, unlike methods such as weighted pruning, which require specialized software or hardware gas pedals to accelerate inference. It mainly achieves model compression by pruning certain layers or channels of the original network, but this also leads to the problem of less flexibility. Its effectiveness is not significant until the network is deep enough. Relative to other techniques, channel-wise sparsity strikes a balance between flexibility and ease of implementation, as it can be applied to any typical CNN or fully connected network, generating results that are essentially simplified versions of the original network.

**Figure 7:**
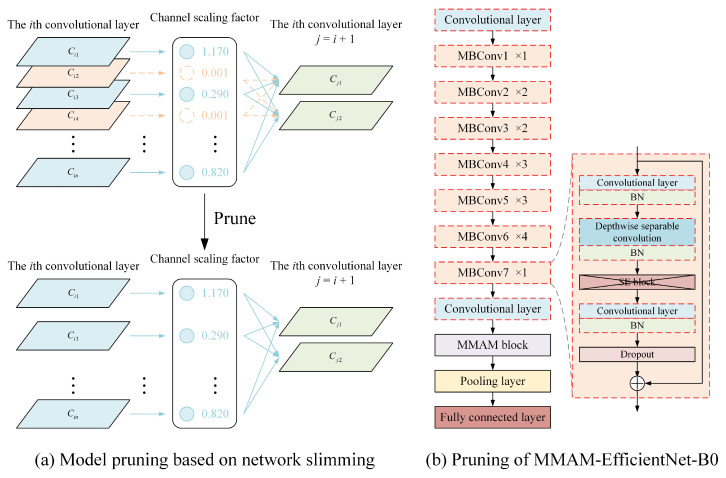
Pruning of MMAM-EfficientNet-B0 based on Network Slimming.

Our method is guided by Network Slimming [11], which crops the convolutional and BN layers of MMAM-EfficientNet-B0, and the realization is shown in Figure 7a. Figure 7b shows the layers that need to be pruned for this network and the improvements made to the network before pruning. Where the red dashed box is the layer to be pruned, containing the convolutional layer, the BN layer, and the depthwise separable convolutional layer. The deletion of the SE module, on the one hand, reduces the influence of the convolutional layer in the SE module caused by pruning, and on the other hand, it can further streamline the network, and the structure of the MMAM-EfficientNet-B0 model has been described in detail in a previous study [8].

In Network Slimming, each channel adds a γ scaling factor, multiplied by the output of that channel. The network weights and these scaling factors are then trained jointly, and sparse regularization is applied to the latter. Finally, the channels with small scaling factors are directly clipped, and the trimmed network is fine-tuned. Specifically, the overall goal equation for Network Slimming is as follows:

(6) $$L=\sum_{\left(x,y\right)}l\left(f\left(x,W\right),\;y\right)+\lambda \sum_{\gamma \in \Gamma }g\left(\gamma \right),$$

where $\left(x,\;y\right)$ denotes the input and target values for training, *W* denotes the trainable weights, $\sum_{\left(x,y\right)}l\left(f\left(x,W\right),\;y\right)$ denotes the value of the training loss of the CNN, $g\left(\cdot \right)$ is the penalty term imposed on the scaling factor, and λ is the balancing factor of the two terms. L1 paradigms are employed in our experiments to achieve sparsity, i.e., $g\left(s\right)=\left|s\right|$, specifically using subgradient descent as an optimization method for non-smooth L1 penalty terms. By applying an L1 paradigm to a channel, pruning all incoming and outgoing connections of that channel, a streamlined network is directly obtained without resorting to special sparse computation packages. The scaling factor indicates the importance of channel selection, and since the regular term of the scaling factor and the weight loss function work together to perform the optimization, the network can automatically identify unimportant channels and remove them, with little impact on the generalization performance of the network.

Currently, most CNNs include a Batch Normalization (BN) layer, which is a standard method for accelerating network convergence and improving performance. The BN layer performs the transformation operation by normalizing the internal activation values with a small number of statistical properties as follows:

(7) $$\hat{z}=\frac{z_{in}-\mu_{B}}{\sqrt{\sigma_{B}^{2}+\varepsilon }};z_{out}=\gamma \hat{z}+\beta ,$$

where $z_{in}$ and $z_{out}$ are the inputs and outputs of the BN layer, respectively; *B* denotes the current small batch, $\mu_{B}$ and $\sigma_{B}$ are the mean and standard deviation values of the input activation on *B*; γ and β are trainable affine transformation parameters, representing scale and shift, respectively, which provide the possibility of a linear transformation for the normalized activation. We utilize the BN layer in MMAM-EfficientNet-B0 as the scaling factor required for Network Slimming. The biggest advantage of this method is that it does not introduce additional overhead to the network.

With the introduction of the scaling factor regularity term, many of the scaling factors in the generated model converge to zero. Channel pruning is performed by removing channels that have a near-zero scaling factor, eliminating all their incoming and outgoing connections and associated weights. Prune the channel on all layers using a global threshold that is some percentile of all scaling factor values. If a scaling factor of 70% is selected for pruning, then 70% of the channels with lower weights will be pruned. This results in a more compact network with fewer parameters, less runtime memory, and fewer computational operations. Although this may temporarily lead to some loss of accuracy at higher pruning rates, this can be compensated for by fine-tuning the pruned network.

## 4. Experiment and Results

In this section, each of the three parts of data expansion, model pruning, and embedded practical test testing will be described.

### 4.1. NEU-CLS Dataset Expansion

Our NEU-CLS dataset expansion is mainly obtained by StyleGAN3 for generating fake images, the WWNR algorithm for removing water-wave-like noise, and Real-ESRGAN for repairing the details, and the quality of GAN-generated images is judged by the image presentation and quantitative metrics in the experiments.

#### 4.1.1. Experimental Environment Setup and Dataset Introduction

The experiments in this subsection are based on the following hardware and software environments: an Intel(R) Xeon(R) Gold 6258R CPU @2.7GHz processor, 512 G RAM, two NVIDIA Tesla V100s-PCIE GPUs with 32 GB of RAM for each GPU respectively, a Python-3.9 interpreter, a PyTorch-1.11 deep learning library, CUDA-12.0 parallel computing architecture, and Windows 10 operating system.

**Figure 8:**
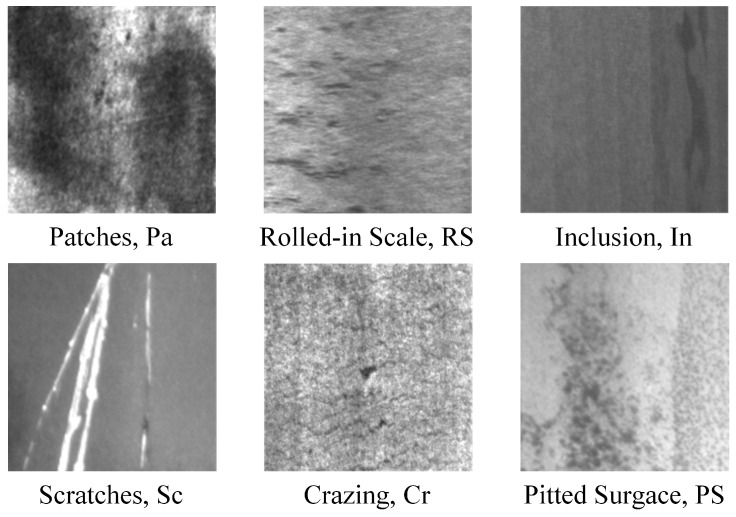
Names and pictures of surface defects on six classes of HRSS in the NEU-CLS dataset.

The dataset used in our experiments comes from NEU-CLS, created by Song et al. [29] at Northeastern University. The dataset contains six classes of HRSS surface defects, as shown in Figure 8, namely Rolled-in Scale (RS) formed by pressing in of secondary iron oxide formed between finishing mills due to roughness of finishing work roll surfaces, Patches (Pa) formed by pressing in of iron filings and other detritus from cracked edges attached to the steel plate during rolling, Crazing (Cr) caused by poor cleaning and overheating of slab edges, Pitted Surface (PS) due to poor management of cooling water in the rolling bath or improper cooling methods, Inclusion (In) due to debris pressed into the steel plate by various rolls attached to the rolling and finishing lines, and Scratches (Sc) due to fixed protrusions on the rolling and finishing lines. There are 300 images of 200 × 200 size for each defect class, and the dataset images are scaled to 224 × 224 × 3 for the convenience of modeling, corresponding to width, height, and channel, respectively.

#### 4.1.2. Data Expansion and Ablation Experiments

**Figure 9:**
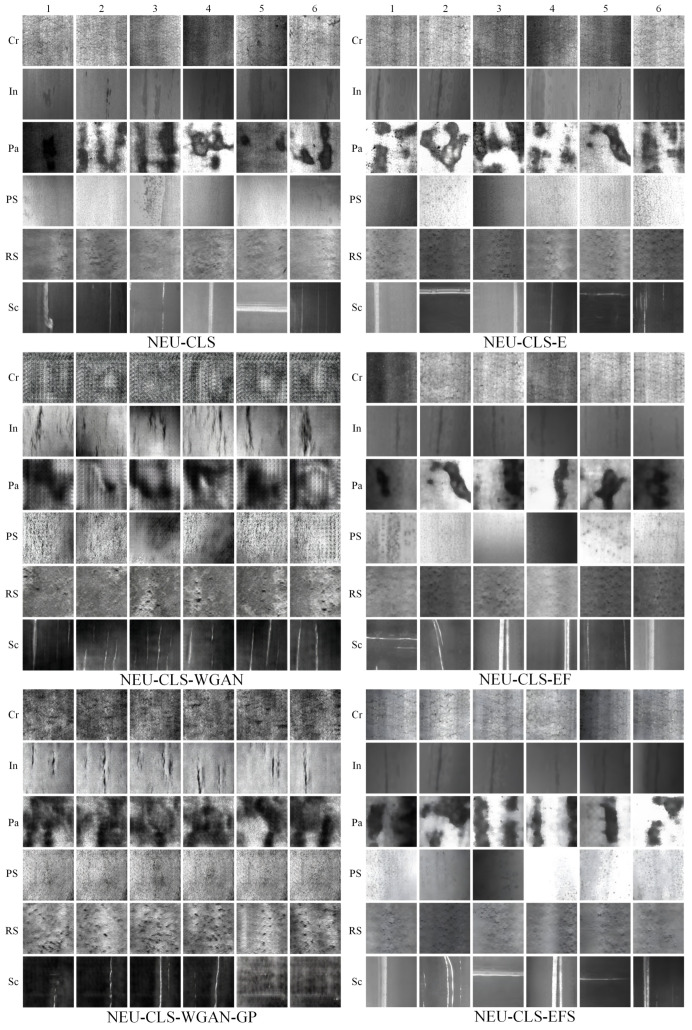
The effect of NEU-CLS data expansion under different methods.

In StyleGAN3’s NEU-CLS image generation experiments for surface defects on HRSS, the learning rates for the generator and discriminator networks are set to 0.002, and the batch size is 32. The gamma is set to 6.6, which is the R1 regularization weight; the larger the value, the more stable the model is, and the smaller the value, the more diverse the model is. The cfg selects StyleGAN3-R mode, i.e., it means that isotropic translations and rotations are included in the generated image. The total number of iterations for a training session is 1000 rounds, and the training duration is 20 h with the support of 2 GPUs. There are six classes of defects in the NEU-CLS dataset, which need to be trained six times to get the generator weights for the six defects. The obtained weights are then used to generate 10,000 images per class, for a total of 60,000 images, named NEU-CLS-E. Then the noise is removed using the WWNR algorithm according to the method in Section 3.2.2 to obtain the NEU-CLS-EF. Finally, NEU-CLS-EF is super-resolved by the trained Resl-ESRGAN network and resized to the original size to obtain NEU-CLS-EFS. To compare the quality of the generated images, WGAN and WGAN-GP were also added in this study, and the generated images were NEU-CLS-WGAN and NEU-CLS-WGAN-GP, respectively. The images generated by these two models and the images generated by StyleGAN3 under the same configuration are shown in Figure 9. It can be seen that the images generated by WGAN and WGAN-GP are mainly dark, with no obvious change in brightness, and there is no StyleGAN3-generated image that has the feeling of natural light and shadow, which appears to be not real enough. And there are obvious jagged lines in Cr, Pa, and PS of NEU-CLS-WGAN, which are seriously inconsistent with the target image. It is moderated in the NEU-CLS-WGAN-GP images, but most of the images in Sc are too dark and the jagged grain is more pronounced in the lighter images. A lot of water-wave-like noise can be seen in the NEU-CLS-E image generated by StyleGAN3, especially In1, In2, and Sc2 are very obvious. With the WWNR algorithm, the water-wave-like noise in the image is reduced significantly, and the details are repaired using Real-ESRGAN to obtain the final dataset NEU-CLS-EFS.

Compared to the original dataset, to the naked eye, there is very little difference in either light and shadow or generation detail. Of course, it is not possible to evaluate the dataset solely with the naked eye, and we use the MMAM-EfficientNet-B0 model from a previous study [8], and the weights trained to achieve 100% accuracy to evaluate images generated by WGAN, WGAN-GP, and StyleGAN3. Each generative model generates 10,000 images per class of defects, for a total of 5 × 6 × 10,000 images, plus the common generative image metric FID (Frechet Inception Distance), to obtain the experimental results shown in Table 1. PV > 0.9 (Predicted Value) denotes that 10,000 defective images generated are recognized as such by the MMAM-EfficientNet-B0 model with weights, and the predicted value is greater than 0.9. UPV (Usable Predicted Value) denotes the average of all PVs recognized as PV > 0.9, and APV (Average Predicted Value) denotes the average of all 10,000 image-recognized PVs. The FID metric measures the distance between the generated samples and the real samples, with smaller values indicating that the generation quality is closer to the original dataset. From the data, our method is not always the best in terms of quality metrics for generating each class of defective images, but these metrics do not fully account for the strengths and weaknesses of the dataset generation, especially the FID values. Currently, the metrics for generated images are evaluated using weights trained on the ImageNet dataset with the Inception V3 network as the recognition network. However, the dataset images used for the experiments are not contained within the ImageNet dataset, so the results of the evaluation are not very accurate.

**Figure 10:**
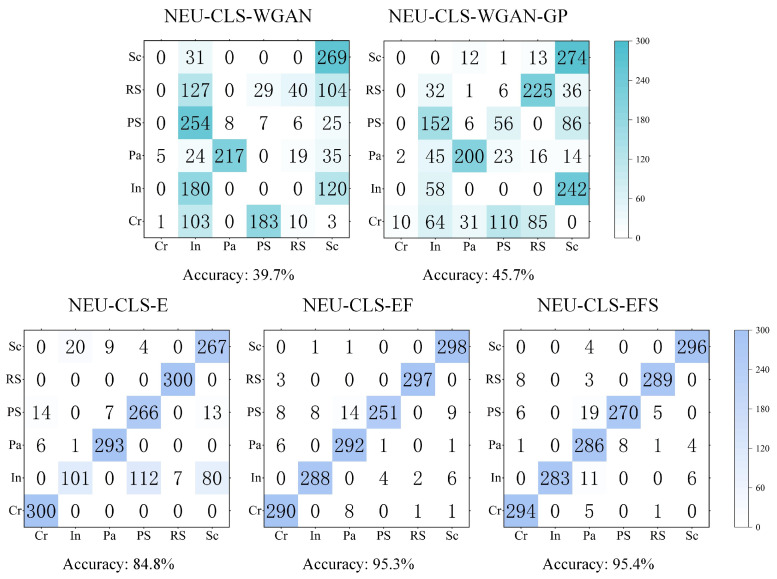
Comparison of image quality of five generated datasets.

Based on this, we test the quality of the generated data by using the generated images instead of the original dataset for training; i.e., the generated images serve as the training set, and the original images as the test set. 1000 images of each class of defects generated in this experiment are selected, and a total of 6000 images for each dataset to be tested are used as MMAM-EfficientNet-B0 training sets, respectively. The dataset to be tested is divided into 8:2 according to the training set and test set, and the stochastic gradient descent (SGD) optimizer is selected, with an initial learning rate of 0.01, decreasing learning rate by cosine annealing algorithm, with momentum set to 0.9, weight decay of 10-4, and a batch size of 4, and training is carried out for 50 epochs. Train according to the same experimental parameters to get the trained network weights and test the original dataset NEU-CLS by the network paired with the weights, and the obtained experimental results are shown in Figure 10:

The figure shows that the generated image of WGAN trained as a training set is very poor in recognizing the NEU-CLS image, except for the Sc and Pa class defects, which are relatively well recognized; the other class defects are very poorly recognized, and the total recognition accuracy is only 39.7%. The recognition performance of the network on Cr, In, and PS types of defects is relatively poor after training on the dataset generated by the WGAN-GP method, with an accuracy of 45.7%. After training on a dataset generated directly by StyleGAN3, recognition performance for defects is significantly improved, with an accuracy of 84.8%, except for the In defect, which still shows relatively poor recognition performance. After denoising by the WWNR algorithm, the recognition of defects in each class is dramatically improved, and the recognition accuracy is increased to 95.3%. Finally, after training the NEU-CLS-EFS dataset obtained from the Real-ESRGAN supplemental details, the defect recognition for each class is further improved, achieving 95.4% recognition accuracy. It can be seen that the training effect of the NEU-CLS-EFS dataset obtained after the method of Section 3.2 in MMAM-EfficientNet-B0 is very close to that of the original dataset NEU-CLS, and also, therefore, the method is very effective for the data expansion of NEU-CLS.

### 4.2. MMAM-EfficientNet-B0 Model Pruning

The pruning to implement the MMAM-EfficientNet-B0 model implements the pruning of the EfficientNet-B0 model. In the network model of EfficientNet-B0, the main part is composed of 16 MBConv modules with different parameters, and the Network Slimming method focuses on pruning the convolutional, depthwise separable convolutional, and BN layers in this module.

#### 4.2.1. Experimental Environment Setup

The experiments in this subsection are based on the following hardware and software environments: an Intel 12-core Xeon E5 processor with 8 G RAM, an Nvidia RTX 3070 Ti GPU with 8 G of graphics memory, the Python-3.7 interpreter, the PyTorch-1.7 deep learning library, the CUDA-11.3 parallel computing architecture, the cuDNN-8.2 deep neural network library, the PyTorch-Lightning acceleration framework version 1.1.5, Windows 10 operating system.

#### 4.2.2. Comparison of Multi-Model Pruning Experiments

A set of controlled experiments is designed to investigate the effect of removing the SE attention module from the EfficientNet-B0 and MMAM-EfficientNet-B0 models on recognition accuracy and model lightweight. These two original models and the model with the SE module removed are trained on the dataset separately, and the accuracy, Loss value, number of parameters, number of floating point computations per second (FLOPs), and training duration of the models are recorded.

**Figure 11:**
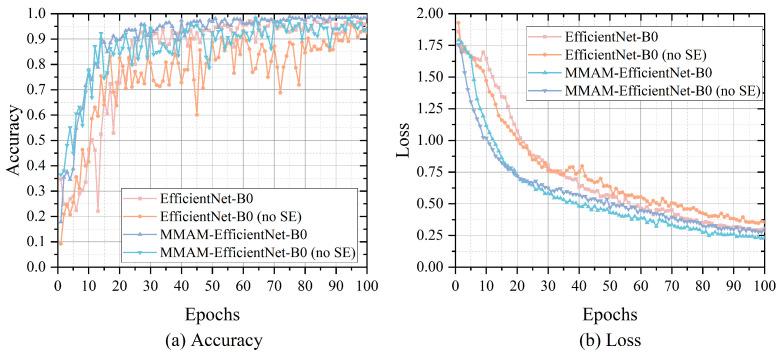
An efficientNet-B0 model with and without SE block-trained results.

In the experiments, the expanded dataset NEU-CLS-EFS obtained in Section 4.1 is divided into 8:2 according to the training set and the test set, and preprocessing operations such as random cropping, flipping, rotating, and erasing are performed. The SGD optimizer is selected, the initial learning rate is set to 0.01, the learning rate is decayed according to the cosine annealing algorithm, the momentum is set to 0.9, the weight decay is set to 10-4, and the batch size is set to 2 for 100 epochs of training. The accuracy and Loss value versus epoch curves of their experimental results are shown in Figure 11. From the figure, it can be seen that the MMAM-EfficientNet-B0 model converges significantly faster than EfficientNet-B0 on the extended NEU-CLS dataset, and the recognition accuracy is also higher. On the comparison of the results with and without the SE module, the EfficientNet model without the SE module converges relatively fast, but due to the poorer convergence values of accuracy and Loss values than the model with the SE module, it results in catching up at a later stage and produces intersections, which is particularly evident in Figure 11b. In terms of the stability of the training results, the network with the SE module gets smoother accuracy per epoch, while the stability of the network without the SE module is not as strong. As shown in Table 2, the effect of the EfficientNet-B0 model with and without the SE module on the number of network parameters, computational complexity, and training duration is demonstrated. Where the accuracy and loss values are averaged over the last 10 epochs of training taken, and the results of FLOPs are calculated with inputs of [1, 3, 224, 224] ([batch size, channel, width, height]).

From the data in the Table 2, it can be seen that the same model without an SE module network decreases the accuracy by about 3 percentage points compared to the original model, the Loss goes up by about 0.06, the parameters decrease by 15.92%, the FLOPs decrease by 0.15%, and the training duration is shortened by 50%. The experimental results show that in the EfficientNet-B0 and MMAM-EfficientNet-B0 models, the lack of an SE module does not have a significant effect on the accuracy, but it greatly reduces the training time and improves the training efficiency of the models. Therefore, the networks with SE modules in the following experiments have the SE modules removed.

**Figure 12:**
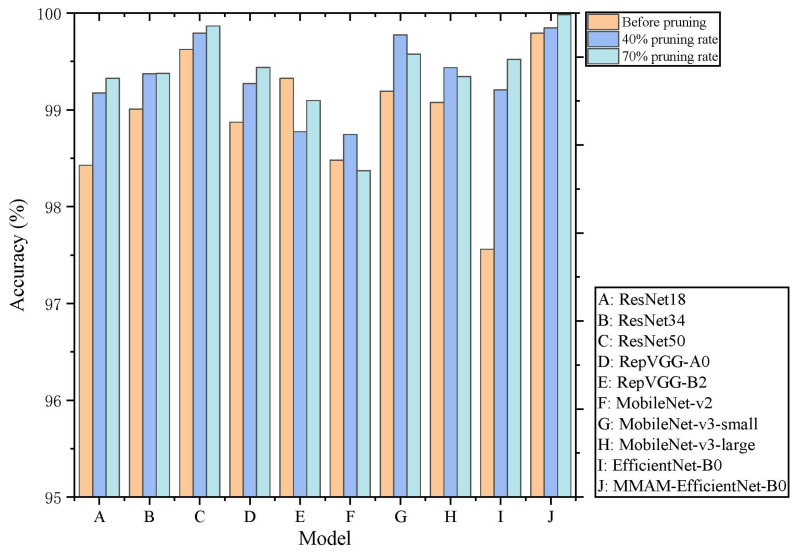
Accuracy of each model at different pruning rates.

The multiple networks selected in this subsection include ResNet18, ResNet34, ResNet50, RepVGG-A0, RepVGG-B2, MobileNet-v2, MobileNet-v3-small, MobileNet-v3-large, EfficientNet-B0, MMAME-fficientNet-B0, totaling 10 common network structures. In the experiments, the learning rate for normal training is set to 0.01, and 120 epochs are trained; the learning rate for fine-tuning training is set to 0.001, and 120 epochs are also trained. The L1 regularization factor is 0.0001, and the batch size is 4. Model training, pruning, and fine-tuning are performed on the NEU-CLS-EFS dataset using the original model, as well as models pruned by 40% and 70%, as shown in Table 3. It can be seen that the recognition accuracy of most models after pruning and fine-tuning has not been reduced, and in some cases has even improved. Channel-level pruning of the convolutional, depthwise separable convolutional, and BN layers results in varying degrees of reductions in the model’s parameter count and computational cost. Among them, MMAM-EfficientNet-B0 has the highest recognition accuracy on the NEU-CLS-EFS dataset among the compared models at different pruning rates, as shown in Figure 12, and the number of parameters and computational effort is relatively low.

#### 4.2.3. Experiments on the Relationship Between Pruning Rate and Changes in Accuracy Before and After Pruning

**Figure 13:**
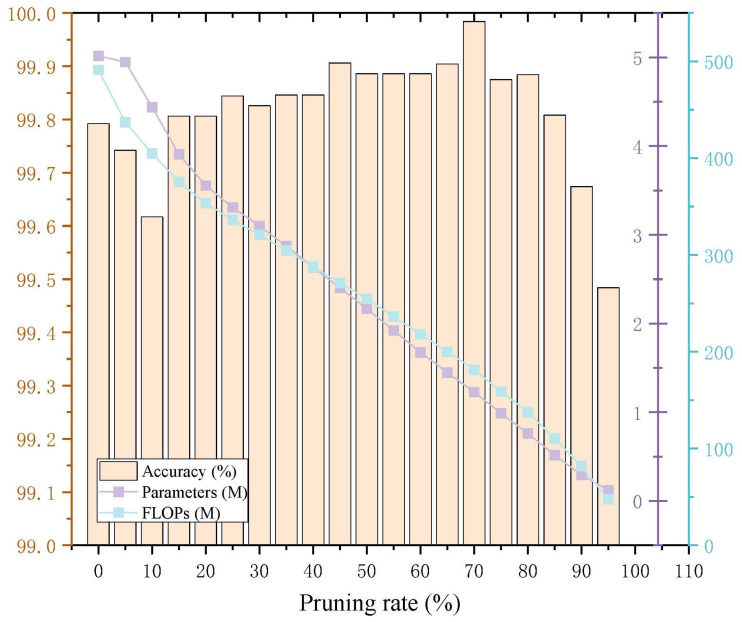
MMAM-EfficientNet-B0 accuracy, parameters, and FLOPs pruning effects.

To select the appropriate pruning rate for model compression of the MMAM-EfficientNet-B0 model, the model is set to be pruned every 5 percentage points. The experimental parameters are the same as in the previous subsection, and the results are shown in Table 4. It can be seen that the MMAM-EfficientNet-B0 model achieves high accuracy across different pruning rates, and its parameters and FLOPs decrease as the pruning rate increases. Figure 13 shows the pruning effects of MMAM-EfficientNet-B0. It can be seen that the accuracy of MMAM-EfficientNet-B0 increases slowly and then decreases sharply with the pruning rate. The parameters and FLOPs decrease slowly, and the accuracy reaches a maximum at a 70% pruning rate. It can be seen that the MMAM-EfficientNet-B0 model works best when the pruning rate reaches 70%.

### 4.3. Raspberry Pi Practical Test Experiment

All the above experiments are implemented on a computer with Windows OS using a GPU, to further demonstrate the recognition and operation effects on the embedded side. In this subsection, we experiment with the recognition and compression effect of the MMAM-EfficientNet-B0 model by Raspberry Pi with a camera.

#### 4.3.1. Raspberry Pi-Based Environment Setup

The experiments in this subsection are based on the following hardware and software environments: a Raspberry Pi 4B with an 8G motherboard with a Logitech camera, a 64-bit Raspberry Pi operating system (Linux), the PyTorch 1.7 deep learning library, and the PyTorch Lightning 1.4.9 acceleration framework.

#### 4.3.2. MMAM-EfficientNet-B0 Recognition and Compression Experiment on Embedded Side

**Figure 14:**
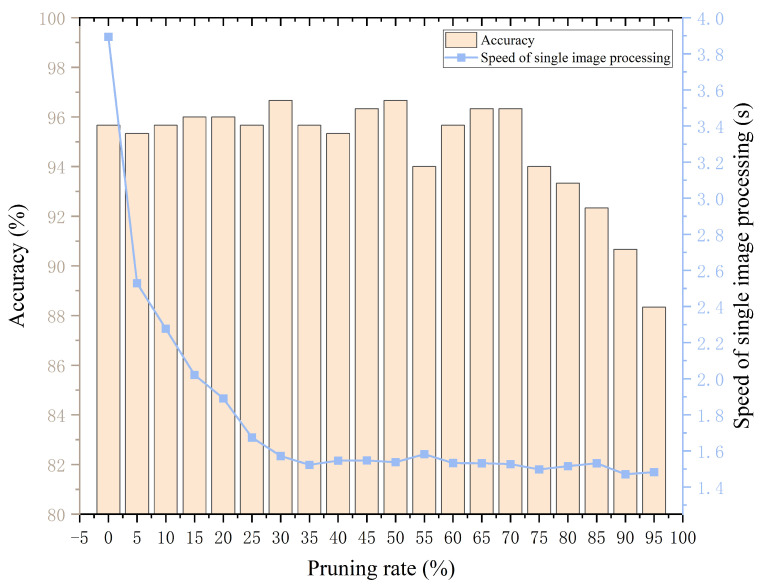
MMAM-EfficientNet-B0 compression on Raspberry Pi.

In this subsection, 50 images of each defect of the NEU-CLS dataset are selected, for a total of 300 images for the practical test experiments on the Raspberry Pi. We take pictures with the camera during the detection process, and then perform identification and analysis on them. The MMAM-EfficientNet-B0 model with different pruning rates is imported sequentially to recognize these 300 images, and the average time and recognition accuracy for recognizing a defective image are calculated as shown in Figure 14. From the figure, we can see the results of MMAM-EfficientNet-B0 with different pruning rates on a Raspberry Pi, including recognition accuracy and the average execution speed per image. Where the recognition speed of a single image decreases relatively fast until the pruning rate reaches 30%, after which it is based on smoothness. The recognition accuracy is around 96% until the pruning rate reaches 70%, after which the accuracy declines more rapidly. To ensure that the model is lightweight enough while guaranteeing recognition accuracy, we choose MMAM-EfficientNet-B0 at a 70% pruning rate, at which time the accuracy is 96.333%, and the recognition and classification time for a single image is 1.527 s, which is reduced by 155.010% compared to the original model. This shows that the model compression method based on Network Slimming is quite effective for MMAM-EfficientNet-B0.

## 5. Conclusions

This paper proposes a lightweight model for detecting surface defects in hot-rolled steel strips. We perform channel-level pruning of the MMAM-EfficientNet-B0 model by removing the SE module inside the model; as a result, we retain 30% of the backbone network. For data expansion, we employ StyleGAN3 to generate fake images, the WWNR algorithm to remove waterline noise, and Real-ESRGAN to repair the details. Finally, in the practical test on Raspberry Pi, a recognition accuracy of 96.333% is obtained, and the recognition and classification time for a single image is 1.527 s, which is reduced by 155.010% compared to the original model. Our work aims to alleviate some of the limitations of existing hot-rolled strip steel detection systems, which rely on complex, costly equipment, are cumbersome to operate, and struggle to meet real-time requirements. They also seek to reduce the computational burden of deep neural networks in real-time detection and large-scale data processing. In the future, we plan to extend our approach to include the classification of a wider variety of steel surface defects, beyond just hot-rolled steel strips. By broadening the scope to encompass more defect types rather than limiting it to the six categories currently addressed, we aim to make our method more general and robust, thereby improving its applicability across diverse industrial scenarios.

## Figures and Tables

**Table 1:** Image generation quality metrics.

Defect Class Name	Generated Image Name	PV > 0.9 (%)	UPV	APV	FID
Cr	NEU-CLS-WGAN	5.70	0.9389	0.6667	259.59
	NEU-CLS-WGAN-GP	32.80	0.9503	0.8026	106.32
	NEU-CLS-E	92.00	0.9933	0.9740	85.87
	NEU-CLS-EF	35.58	0.9716	0.8118	106.78
	NEU-CLS-EFS	29.28	0.9700	0.7572	154.35
In	NEU-CLS-WGAN	99.80	0.9987	0.9984	279.73
	NEU-CLS-WGAN-GP	98.40	0.9962	0.9933	125.19
	NEU-CLS-E	7.05	0.9500	0.7490	338.36
	NEU-CLS-EF	25.81	0.9501	0.7900	206.70
	NEU-CLS-EFS	54.36	0.9627	0.8576	162.53
Pa	NEU-CLS-WGAN	66.00	0.9671	0.8893	217.34
	NEU-CLS-WGAN-GP	100.00	0.9977	0.9977	167.87
	NEU-CLS-E	81.23	0.9800	0.9388	245.56
	NEU-CLS-EF	13.00	0.9508	0.6869	186.16
	NEU-CLS-EFS	15.40	0.9489	0.7172	258.22
PS	NEU-CLS-WGAN	96.30	0.9962	0.9845	130.34
	NEU-CLS-WGAN-GP	88.20	0.9722	0.9552	169.05
	NEU-CLS-E	94.83	0.9896	0.9780	238.66
	NEU-CLS-EF	77.26	0.9750	0.9160	101.90
	NEU-CLS-EFS	6.09	0.9545	0.5866	202.70
RS	NEU-CLS-WGAN	86.40	0.9863	0.9537	96.56
	NEU-CLS-WGAN-GP	100.00	0.9998	0.9998	100.39
	NEU-CLS-E	91.36	0.9916	0.9689	119.69
	NEU-CLS-EF	40.44	0.9664	0.8185	151.93
	NEU-CLS-EFS	42.64	0.9665	0.8180	184.63
Sc	NEU-CLS-WGAN	99.60	0.9962	0.9955	214.83
	NEU-CLS-WGAN-GP	95.10	0.9898	0.9785	242.96
	NEU-CLS-E	98.16	0.9970	0.9915	248.20
	NEU-CLS-EF	98.39	0.9963	0.9913	146.50
	NEU-CLS-EFS	98.08	0.9949	0.9895	172.00

**Table 2:** EfficientNet-B0 model with and without SE module lightweight results.

Network Model	Accuracy (%)	Loss	Parameter (M)	FLOPs (G)	Training Duration
EfficientNet-B0	95.308	0.3024	4.02	411.56	6 h 7 min 32 s 442 ms
EfficientNet-B0 (no SE)	92.108	0.3620	3.38	410.94	3 h 5 min 25 s 40 ms
EfficientNet-B0-MMAM	98.263	0.2276	5.66	491.84	6 h 6 min 51 s 40 ms
EfficientNet-B0-MMAM (no SE)	95.125	0.2920	5.02	491.22	3 h 30 min 39 s 911 ms

**Table 3:** The results of comparison of multi-model pruning experiments.

Model Name	Accuracy (%)	Parameters (M)	Parameters Pruning Rate (%)	FLOPs (G)	FLOPs Pruning Rate (%)
ResNet18 (Baseline)	98.428	11.172	-	27.336	-
ResNet18 (40% pruned)	99.174	7.259	35.025	14.243	47.897
ResNet18 (70% pruned)	99.326	1.803	83.861	5.973	78.150
ResNet34 (Baseline)	99.008	21.280	-	57.012	-
ResNet34 (40% pruned)	99.374	13.775	35.268	32.785	24.227
ResNet34 (70% pruned)	99.376	4.247	80.042	15.975	71.980
ResNet50 (Baseline)	99.624	23.513	-	64.267	-
ResNet50 (40% pruned)	99.792	11.763	49.972	28.770	55.234
ResNet50 (70% pruned)	99.866	4.857	79.343	17.473	72.812
RepVGG-A0 (Baseline)	98.872	7.836	-	22.422	-
RepVGG-A0 (40% pruned)	99.272	7.704	1.685	22.084	1.507
RepVGG-A0 (70% pruned)	99.437	6.571	16.143	21.019	6.257
RepVGG-B2 (Baseline)	99.326	86.477	-	294.388	-
RepVGG-B2 (40% pruned)	98.774	86.150	0.378	293.159	0.417
RepVGG-B2 (70% pruned)	99.096	82.919	4.114	285.854	2.889
MobileNet-v2 (Baseline)	98.481	2.232	-	51.515	-
MobileNet-v2 (40% pruned)	98.745	1.267	43.235	37.313	27.569
MobileNet-v2 (70% pruned)	98.372	0.609	72.715	19.640	61.875
MobileNet-v3-small (Baseline)	99.192	1.062	-	0.888	-
MobileNet-v3-small (40% pruned)	99.774	0.901	15.160	0.559	37.049
MobileNet-v3-small (70% pruned)	99.575	0.654	38.701	0.308	65.315
MobileNet-v3-large (Baseline)	99.076	2.697	-	3.338	-
MobileNet-v3-large (40% pruned)	99.436	2.099	22.173	1.987	40.473
MobileNet-v3-large (70% pruned)	99.344	1.368	49.277	0.943	71.750
EfficientNet-B0 (Baseline)	97.560	3.379	-	0.411	-
EfficientNet-B0 (40% pruned)	99.206	2.667	21.071	0.333	18.978
EfficientNet-B0 (70% pruned)	99.522	1.185	64.930	0.161	60.827
MMAM-EfficientNet-B0 (Baseline)	99.792	5.017	-	0.491	-
MMAM-EfficientNet-B0 (40% pruned)	99.846	2.635	47.479	0.288	41.415
MMAM-EfficientNet-B0 (70% pruned)	99.984	1.227	75.543	0.181	63.058

**Table 4:** Comparison of accuracy, parameters, and FLOPs under different pruning rates of MMAM-EfficientNet-B0.

Pruning Rate (%)	Accuracy (%)	Parameters (M)	Parameters Pruning Rate (%)	FLOPs (G)	FLOPs Pruning Rate (%)
Baseline	99.792	5.017	-	491.212	-
5	99.742	4.948	1.375	437.240	10.988
10	99.617	4.439	11.521	404.973	17.556
15	99.806	3.907	22.125	375.600	23.536
20	99.806	3.555	29.141	353.492	28.037
25	99.844	3.310	34.024	336.288	31.539
30	99.826	3.098	38.250	320.776	34.697
35	99.846	2.874	42.715	304.718	37.966
40	99.846	2.635	47.479	287.776	41.415
45	99.906	2.402	52.123	270.936	44.843
50	99.886	2.167	56.807	254.798	48.129
55	99.886	1.921	61.710	236.279	51.899
60	99.886	1.676	66.594	218.130	55.594
65	99.904	1.449	71.118	200.110	59.262
70	99.984	1.227	75.543	181.465	63.058
75	99.875	0.989	80.287	158.901	67.651
80	99.884	0.762	84.812	137.801	71.947
85	99.808	0.516	89.715	110.326	77.540
90	99.674	0.294	94.140	81.615	83.385
95	99.484	0.120	97.608	47.530	90.324

## Data Availability

The NEU-CLS dataset used in this work can be downloaded from the Google repository: https://drive.google.com/file/d/1NGlXT9sIaQpyxUoT6MLKm1Pr6x8oxOvc/view, accessed on 2 March 2026.

## Abbreviations

The following abbreviations are used in this manuscript:

HRSS	Hot-Rolled Steel Strip
WWNR	Water-Wave-like Noise Removal
SE	Squeeze-and-Excitation
GAN	Generative Adversarial Network
RRDB	Residual-in-Residual Dense Block
SGD	Stochastic Gradient Descent
